# Patient perspectives on noninvasive prenatal testing among black women in the United States: a scoping review

**DOI:** 10.1186/s12884-023-05423-w

**Published:** 2023-03-16

**Authors:** Shameka P. Thomas, Madison A. Keller, Tiara Ranson, Rachele E. Willard

**Affiliations:** 1 School of Public Health, Harvard University, Cambridge, USA; 2 National Institutes of Health-National Genome Research Institute, Bethesda, USA; 3 Louisiana State University-School of Medicine, Baton Rouge, USA; 4 University of Washington-Seattle, Seattle, USA

**Keywords:** NIPT, Cell-free DNA, Black women, Pregnancy, Patient-perspectives, United States

## Abstract

Advances in reproductive health technologies such as noninvasive prenatal testing (NIPT) are changing the landscape of prenatal care and maternal health. NIPT, made clinically available in the United States (US) in 2011, is a screening test that utilizes cell-free DNA (cfDNA) to detect for aneuploidies and genetic characteristics in fetal DNA. In September 2020, the American College of Obstetricians and Gynecologists (ACOG) recommended NIPT for all pregnant patients regardless of age or risk factors. We examined peer-reviewed, empirical studies published from January 2011 to February 2022, assessing NIPT studies with patient perspectives in the US and what is known about how empirical studies include Black women. Our scoping review draws from PubMed (with advanced MeSH search options) and Scopus databases for advanced scoping review, with 33 articles meeting our criteria. Empirical studies on NIPT show patient perceptions range across five themes: 1) accuracy / safety, 2) return of results, 3) patient knowledge, 4) informed consent, and 5) perceptions among minoritized groups (with perceptions of race and gender as a social demographic intersection). Additionally, among the 15 studies that included that Black woman in their study sample, none measured the perceptions of Black women with genetic conditions. Bridging this knowledge gap is critical because NIPT is becoming increasingly accessible across the nation and is being developed to screen for additional genetic conditions, such as sickle cell disease. Ultimately, NIPT researchers need to go to greater lengths to examine the patient perspectives of Black women with and without genetic conditions.

## Background

Advances in reproductive genetic technologies such as non-invasive prenatal testing (NIPT) are changing the landscape of prenatal healthcare in the United States (US) and across more than 50 countries [1, 2]. NIPT, made commercially and clinically available in the US in 2011, is a novel genetic screening test utilized for detecting aneuploidies and genetic characteristics in fetal DNA (e.g., sex chromosomal characteristics and / or trisomy 21, 13, and 18). Developers of NIPT are currently testing approaches using cell-free fetal DNA to screen for additional genetic conditions, such as sickle cell disease (SCD) [3, 4]. NIPT’s capability to screen for these genetic conditions would reduce the over-utilization of invasive tests (e.g., amniocentesis and chorionic villus sampling) which are used to diagnose certain genetic conditions. Invasive tests are not only higher in costs, but also associated with increased susceptibility to procedure-related pregnancy-loss [3, 5]. As of September 2020, the American College of Obstetricians and Gynecologists (ACOG) recommended NIPT as standard, routine prenatal care in the US to reduce reliance on invasive prenatal tests and because it is deemed as clinically beneficial for pregnant people of both high-risk and low-risk prenatal status [2].

Now more than ever, it is the time to ask critical questions about how researchers are examining the patient perspectives of NIPT in the US. Our guiding premise was to investigate which aspects of empirical-based NIPT studies researchers include when assessing patient perspectives, with an intentional focus on minoritized populations such as Black women. Black women are identified as a historically vulnerable group in the US due to intersecting social factors, such as racism, sexism, and class-based discriminations [6, 7]. Thus, our research scope is two-part: 1) an assessment of patient perspectives from empirical studies; and 2) an evaluation of how researchers examine the demographic of Black women across NIPT study samples. We consider these two-parts as interconnected, particularly since understanding empirical studies on a national level is contingent to measuring how researchers investigate marginalized demographics.

The relevance for this investigation, specifically regarding who (or what) is missing across empirical-based NIPT studies, is the key factor that distinguishes our scoping review in the field of prenatal genetic technology and reproductive medicine. A recent qualitative review on NIPT perspectives among pregnant people, family members, and partners showed that majority agree with NIPT utilization as a prolific technology and thus appreciate NIPT’s safety compared to invasive tests [1]. Findings further showed that women also expressed dissatisfaction with how NIPT knowledge is disseminated during clinical encounters. In this regard, patient’s concerns were related to the limited details in the informed consent process for NIPT routinization prior to consenting to participating in its usage [8]. Though both these studies were substantive, they only had results from majority White women in highly-educated groups from high-income countries. Thus, we are curious to know what results such as these would mean for historically vulnerable populations, such as Black women, who are also navigating a high-income country like the US, but generally have less access to quality reproductive healthcare across the national and state levels [6].

Since there has not been a single US-based scoping review that captures how studies incorporate Black women’s perspectives of NIPT, this study sought to bridge that gap. This is necessary because pregnant women often express having anxiety after receiving NIPT results, which are merely predictive (e.g., false-positive or false-negative) and receiving these NIPT results in the early stages of pregnancy was also found to be a contributing factor in adverse prenatal care experience [9]. Farrell and colleagues (2014) conducted a qualitative study on NIPT perspectives (which included a high percentage of Black women in their study sample) and found that patients desire additional NIPT education on the return of results, specifically in terms of uptake, cost of second opinions, and insurance coverage. Thus, we aim to explore the variance among Black women (as a non-monolithic group) by aiming to dive deeper into the types of Black women incorporated into empirical-based NIPT studies at the national level.

## Methods

Our scoping review utilizes the Joanna Briggs Institute (JBI) guideline and the PRIMSA extension of scoping reviews [10–13]. The PRISMA extension of scoping reviews (PRISMA-ScR) guided our checklist [12], coupled with the JBI guidelines. The JBI has nine tenets for conducting a scoping review, which include: identifying the research question, defining the inclusion and exclusion criteria, developing the search strategy (e.g., routes for study selection, data extraction, and evidence), evidence selection, evidence analysis, evidence extraction, presentation of results, summary of the search process, and establishing conclusions as it relates to addressing the study aims [13].

### Eligibility criteria and information sources

**Fig. 1:**
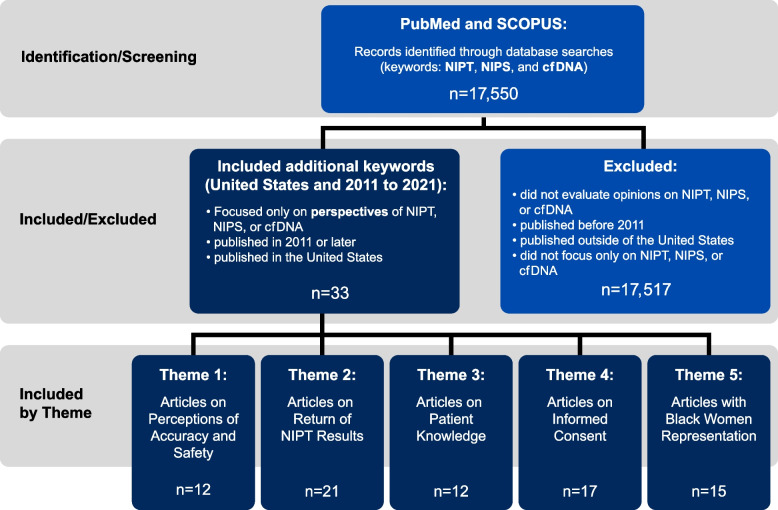
Flow Chart of the Electronic Search Strategy of Empirical Articles on Patient Perspectives on NIPT (with regard to racial demographics) in the United States, 2011–2022

PubMed (with advanced MeSH search options) and Scopus databases were thoroughly searched to access empirical-based NIPT studies (see Fig. 1). Our rationale for using these two databases is that PubMed® includes biomedical literature from MEDLINE, life science journals, and online books with more than 34 million citations and is the primary medical literature database. Scopus is an interdisciplinary database that includes more than 240 disciplines, including social scientific based studies that can speak to non-clinical studies on patient perceptions of noninvasive prenatal testing. We recognize that a limitation of the study is that other databases could be searched, however we believe these two databases will capture most of the articles. The date restriction was set between January 2011 (the year NIPT was first commercially advertised and clinically utilized in the US) to January 2022.

The Boolean string of our key words were “NIPT” OR “NIPS” OR “cell-free-DNA” OR “perspectives.” These words were broadly related to NIPT, so as not to miss any relevant empirical-based studies. Then we set an exclusion to extract all non-US studies from our broad keyword search strategy.

### Specific electronic search strategy

As per PRISMA guidelines [12], our complete search strategy is listed in step-by-step process. Step one focuses on our research aim, which our research team had several analytical discussions to narrow our objections. Step two involved the identification and screening process of the pertinent studies. Step three draws upon a standardized review for charting the data. Below we describe our steps in detail.

### Step 1: Research Aim

Prior to developing our research question and study aims, our scoping review was based on a series of inductive observations that guided our specific electronic search strategy. Our research questions involved: 1) What is known about the patient perspectives of empirical NIPT studies at the national level in the US? and 2) What is known about the demographics of Black women included in those empirical NIPT studies, if so? We divided our study aims into two parts to address these questions. Part one aimed to examine the types of patterns that NIPT researchers most frequently identify as the key themes at the national level. Part two aimed to examine if there are any Black women included in these studies, and if so, what is known (e.g., demographics) about the Black women who are included. Thus, part two of our aims was the centerpiece of our scoping review to provide a robust comparable analysis to part one’s inquiry.

### Step 2: Identification of Relevant Studies

The identification of relevant studies was conducted through two search engines involving PubMed and Scopus with MeSH advanced search options. The screening process of the title and abstract were conducted in duplicate, involving three researchers (ST, MK, and TR) and the senior research library at our institution. Phase one involved review of both the title and abstract. Phase two involved the full reading of the article if it met our screening parameters. We narrowed our search using a Boolean string of key words specific to *NIPT*, *NIPS* and *cfDNA* (acronyms available after references).

Figure 1 illustrates our scoping review utilizing these systematic search options. We began with our search in PubMed and SCOPUS. We restricted our key word search to three terms *NIPT, NIPS, and cfDNA* which yielded 17, 550 total articles. Based on this total, we excluded all articles that were non-empirical (e.g., commentaries), published before 2011, published outside of the US, and articles that did not focus on NIPT, NIPS, or cfDNA. Inclusion was based on empirical articles that focused solely on perspectives of NIPT, NIPS, and cfDNA published after 2011 in the US. We yielded 33 total articles based on these inclusion and exclusion parameters. Within these 33 articles, we excluded articles that did not include race demographics, which left us with 21 total articles. Since our premise was to scope for empirical studies of NIPT that included Black women in their study samples, we then identified 15 of those 21 articles that included Black women.

### Step 3: Charting the Data

Drawing from Arksey and O’Malley’s (2005) steps for scoping review process, we designed a standardized format to chart our data that provides a systematic process for reliability and validity [11]. There are two reasons why we did not specify if these empirical studies are either qualitative or quantitative: 1) some of the studies had approaches of mixed-methods; and 2) we did not want the methodological approach to over shadow the importance of the key themes. Our rationale for this is that the themes are necessary for the field, regardless of either methodological approach. Thus, we did not exclude articles on the basis of their respective methodological approach.

Our review process was divided into two phases. Phase one included screening titles and abstracts. Phase two involved a robust review of each article in EXCEL. We dissected each article across five categories including: study population, aims of the study, methodology, outcome measures, and important results. An additional category was created for capturing what we noticed about what is missing from NIPT studies. We provide a flow chart (see Fig. 1) that illustrates steps one, two and three of our methodology and scoping review process.

## Results

**Fig. 2:**
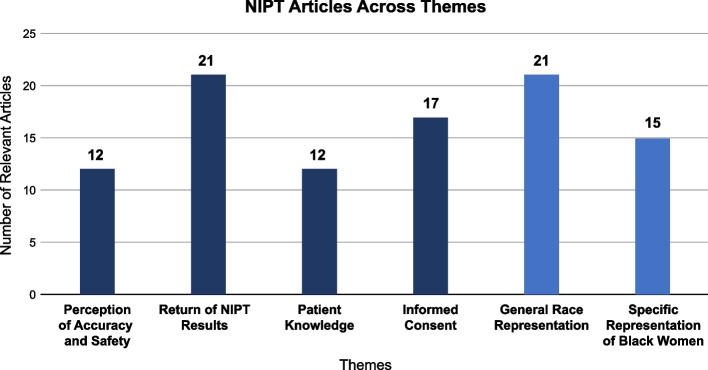
Results of NIPT Articles across Key Themes

Thirty-three empirical studies met our criteria (see Fig. 2); and among these articles, patient perceptions of NIPT ranged across five themes, involving: 1) accuracy and safety, 2) return of results, 3) patient knowledge 4) informed consent, and 5) perceptions among minoritized groups (with perceptions of race and gender as a social demographic intersection) [14–46]. More specifically, the fifth theme showed that although 21 studies included race as a demographic to describe study populations [14–34], only 15 studies highlighted Black women as potential recipients of genetic screening [14–28]. Of these 15 articles, none of those studies measured the perceptions of Black women with genetic conditions, such as sickle cell disease.

### Theme 1: perception of accuracy and safety

Twelve articles discussed patient’s and provider’s views on the accuracy and safety of NIPT [14–22, 29, 35, 36]. Across these articles, NIPT was perceived as safer when compared to invasive methods (e.g., amniocentesis or CVS). NIPT results are predictive. Predictive, in this case, means that NIPT results can only screen for genetic conditions, not determine them definitively [2, 5]. Thus, patient perceptions of accuracy of these predictive test results were viewed as sufficient for uptake of NIPT for all pregnant women, but contingent on patient’s preference.

### Theme 2: return of NIPT results

Twenty-one articles discussed perceptions of NIPT’s return of results [15, 18–25, 29–40]. Of all the articles, this theme was the most significant. Patients expressed both positive and negative views on NIPT results as it relates to clinical decision-making, particularly since the results are predictive. These articles also illustrate that although patients expressed that knowing predictive results (in the first trimester of pregnancy) are beneficial for preparing to have an infant with a potential genetic condition, it also increased stress for pregnant people during the remainder of the pregnancy. Perceptions of negative results were also of concern, due to the results only being predictive, thereby positioning the patient to have a brief timeframe to decide for next steps (e.g., uptake for diagnostic invasive testing and / or early termination of fetus).

### Theme 3: patient knowledge

Twelve of the articles showed results for patients wanting to have increased knowledge of NIPT [14, 22–26, 36, 37, 41–44]. In these articles, patients wanted to know more about how NIPT could mitigate their reproductive health concerns. These articles also showed that there are concerns about how NIPT knowledge is disseminated between both physicians and genetic counselors as well as the standardization of NIPT knowledge. Understanding how patients perceive NIPT knowledge as well as the dissemination of NIPT knowledge is critical for increasing patient-provider communication as well as increasing NIPT uptake. Without critical assessment of what the patient knows about NIPT, no matter how beneficial the NIPT is (or becomes), it can inherently run the risk of being perceived as unethical.

### Theme 4: informed consent

Seventeen of the articles discussed how patients perceived informed consent [16, 19, 21, 25, 27–30, 32–34, 37, 38, 42, 44–46] and how patients receive pertinent details for conducting the screening test. Informed consent is closely related to patient knowledge of NIPT, but it is not a replacement for informed consent. In other words, informed consent and patient knowledge should not be conflated. Informed consent happens *during* the clinical encounter, whereas patient knowledge happens primarily *before* the clinical encounter. Across these articles, however, this key difference was not explicitly stated. This is another critical aspect for advancing the literature surrounding the efficacy of NIPT and informed consent. Ultimately, when there are increased articles on perceptions of informed consent, the next inquiry becomes: who are the patients in these study samples and how nuanced are their baseline knowledge levels compared to the general population?

### Theme 5: perceptions among minoritized groups, emphasizing black women

**Fig. 3:**
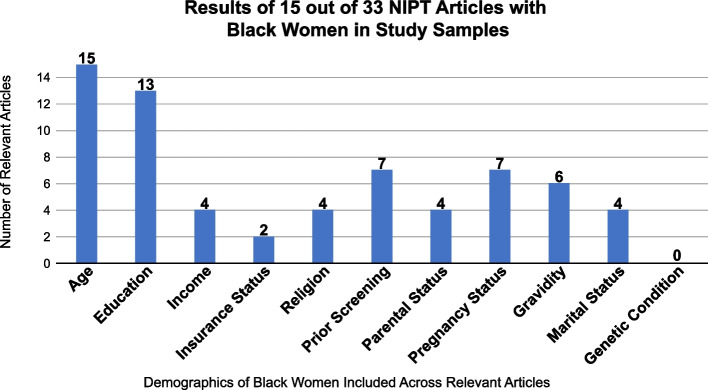
A Tally on the Type of Demographic Information Collected on Black Women in Study Samples in the Included Studies

Unlike most scoping reviews on reproductive genetic technology, our study provides results on the number of empirical articles that included racial demographics among minoritized groups. In this regard, we focused on Black women included in these studies (see Fig. 3). Of the thirty-three empirical articles, 21 articles observe the race of participants [14–34]. However, of those 21 articles, fifteen of the articles included Black women in their study samples as representative of patients and NIPT recipients [14–28], rather than as health professionals (genetic counselors or physicians) providing NIPT and cfDNA screening. We determined the average of Black women represented in the fifteen studies was 12.34%. This percentage was based on comparing the Black subpopulations to overall average of empirical NIPT articles on patient perceptions among women. Please note none of the articles out of that 12.34% specifically clarified the number of study participants who were both Black (self-reported) and female (as self-identifying), and thus we did not want to over-assume.

The fifteen articles showed demographics of the general population (see Fig. 3) that can be appliable to the Black women patient subset population: Age, Education, Income, Insurance, Religion, Prior Screening, Parental Status, Pregnancy Status, Gravidity, Marital Status, and Genetic Condition. All fifteen articles included age as a demographic [14–28]. Thirteen articles included education [14–19, 21, 23–28], but only four articles included income as a demographic [14, 15, 18, 24]. Two articles mentioned insurance as a demographic to describe their sample population [22, 26]. Four articles mention religion as a demographic [14, 15, 23, 24]; four articles included parental status [14, 15, 22, 24], and four articles included marital status as demographics of the general population [16, 18, 24, 26]. When it came to painting a picture of the women undergoing prenatal screening - incorporating pregnancy related demographics – seven articles included pregnancy status [16, 17, 19, 21, 22, 25, 27], six articles included gravidity [16, 19–21, 27, 28], and seven articles included prior NIPT screening as a demographic characteristic [16, 19, 21, 23, 25, 27, 28]. However, of the fifteen articles that included Black women as a subset population, there were zero articles that observed presence of a genetic condition as a demographic.

## Discussion

Of the 33 articles that met our criteria, fifteen of the articles include Black women in their study samples. However, the challenge is how researchers are going about the data collection process, particularly in the field of health disparities. In other words, these results further challenged how the field views demographic data, not only on national-level NIPT studies, but also *what is known about* how minoritized populations, such as Black women with and without genetic conditions are represented in sample sizes.

Theme five is the most important finding of our research, which revealed that of the fifteen articles, none of the articles discussed Black women with genetic conditions. For example, although Black women’s perspectives on NIPT are included in studies between 2011 and 2022, we identified that Black women (with genetic conditions) are not substantively incorporated into the study samples of NIPT articles. Since NIPT has been made clinically available in the US since 2011 this reveals a critical gap. Meaning, it is a critical gap that literature on NIPT has no data on patient perceptions of NIPT among groups with genetic conditions. In other words, NIPT researchers have neglected to view genetics conditions as a demographic category that can impact not only the lived experience, but also how this limitation of NIPT knowledge misinforms reproductive health discourse. This also raises concerns about how these limitation in the field perpetuates missing data in the field, particularly on NIPT and genetic conditions. Failing to be inclusive of these demographic details further contributes to methodological inequities, such as framing and routinizing NIPT for all pregnant people, while not having sufficient data on patient perceptions among the most marginalized groups of people across the nation.

NIPT studies often disseminate data that is seemingly representative of the national population. However, our results show that this data is largely based on White, female respondents who are over-represented across study samples, with very few empirical studies on the patient perceptions of minoritized populations who have varied lived experiences of comorbidities or pre-existing health conditions. This does not mean that none of the Black women did not have genetic conditions, such as sickle cell disease. Rather, it means that the researchers did not focus on genetic conditions as a demographic category.

This demographic gap perhaps occurred for a variety of reasons, ranging from researchers not being socialized or trained to view a genetic condition as a demographic category or simply due to time or budget constraints. Often times, demographics are conventionally portrayed as collecting characteristics on age, education level, racial / ethnic background, insurance status and so forth. However, one of our key findings is that researchers of NIPT studies also need to collect data on genetic conditions as a demographic category. Collecting data on genetic conditions as a demographic not only broadens research on reproductive inequities, but also it allows for a more nuanced understanding of patient populations. This is particularly important when conducting studies on advanced reproductive genetic technologies, such as NIPT, as well as among Black women who are not a monolithic group. Either way, scoping the research landscape of what is known and unknown about NIPT patient perceptions regarding minoritized groups and Black women with and without genetic conditions remains a major gap in the literature.

For the advancement of reproductive equity, it is critical to connect these inquires to broader social and ethical implications. For example, the strength of our study design provides an opportunity to see how NIPT research is conducted at the national level with women from various groups and backgrounds, while providing comparative data that disentangles Black women from being a mere monolithic category. If we were not intentional about our study design in this way, not only would we miss an opportunity to report on racial gap areas, but also areas of medical gaps (e.g., sickle cell disease) and the impact of these similarities and differences across NIPT studies for a more robust scoping analysis.

The discussion of our results generates more ongoing considerations for the field. For example, how do current US-based studies examine the NIPT perceptions of Black women (with and without genetic conditions), particularly now that ACOG has recommended NIPT as routine prenatal care as of September 2020? Who are the women included in clinical trials for NIPT advancement? Are women (who are susceptible to high-risk pregnancy and / or considered medically vulnerable) sufficiently incorporated into ongoing NIPT studies? If so, do these studies include Black women with genetic conditions, such as sickle cell disease? These inquiries are needed in the field of reproductive health disparities as it relates to genetic and precision medicine.

Expanding the literature on reproductive equity, specifically regarding race, racism, and the advancement of research on NIPT, researchers need to ask themselves two questions: 1) do my demographic variables include “race” and “gender” as separated and / or intersected? and 2) are these categories of “race” and / or “gender” self-reported categories or based on the lived experience of perceived-racial categories (e.g., self-reported Latina, but perceived as Black / Afro-Latina) [47, 48]? Currently used racial demographics have limitations, and to advance understandings of racial equity the call to action must be to disentangle the overly-assumed “race” categories in the field of reproductive health (e.g., self-reported race and/or perceived race). This is important because researchers often utilize racial demographics without careful considerations of the historical and present-day implications that impact the lived experience of race, racism, and gender-based racism.

### Strengths and limitations

The strengths of our study are discovering the lack of inclusion on the intersectional dynamics of reporting racial demographic of Black women. For example, due to the intersectional identity of “Black” and “Women,” it is unclear if the quantitative articles (that did include Black women as study participants) used interaction variables or separate demographic variables for race and gender. In other words, we observed empirical articles that included demographics on race [14–34] and gender [14–25, 27–37, 39, 42, 44], but we did not want to incorrectly assume that these categories were intersected subsamples. In this regard, when it comes to the intersectional aspects of race and gender, researchers must go to more intentional lengths to include Black women as an intersectional demographic. Another strength is the two-part study design that not only allowed for us to measure NIPT at the national level, but also to narrow down our focus to see how researchers go about their study samples on Black women more specifically.

Our study is not without limitations. First, we recognize that our scoping strategy is restricted to two major databases (PubMed and Scopus), as additional databases may have reputable studies on the patient perceptions of NIPT innovation. Also, we acknowledge that there may be NIPT research that is being conducted, but not yet published. For example, the initial (baseline) restriction our search was set between January 2011 and January 2021. Then, there was an eight-month window period between when we conducted our final search between January 2011 to November 2021 (to cross-check our article numbers). Thus, additional articles could have been published in other databases since our baseline search (from fall 2021 to spring 2022). In that window period, however, we noticed no additional articles on NIPT patient perspectives were published, which strengthens the reliability and validity of our article totals. However, even if additional articles were published that capture our study's scope, we argue that it would not be a significant increase in the number of studies to refute our claims. In other words, while more research is needed on the NIPT perceptions of Black women (with and without genetic conditions), the field is still far behind the mark at the national-level.

## Conclusion

Conducting a scoping review on US-based NIPT studies provides a substantial opportunity for the field of reproductive health and genetic technology. Examining Black women’s visibility (or lack thereof) in NIPT studies will help researchers to identify potential areas for intervention and improvements in standard prenatal healthcare, specifically for populations (with genetic conditions) who are underrepresented and vulnerable to structural racism.

While NIPT is a genetic technology for fetal screening, we continue to fail women (particularly those susceptible to institutional marginalization) by neglecting to understand how it effects their reproductive autonomy in the social world [48]. Examining patient perceptions among a wide variety of groups with and without genetic conditions is the effective next step to advance efficacy and expand reproductive health equity.

## Data Availability

This study is a scoping review, thus no datasets were generated or analyzed during the study phase. Therefore, providing details on data repository are not applicable. However, the datasets used and/or analyzed during the current study are available from the corresponding author(s) on reasonable request.

## Abbreviations

NIPT: Noninvasive prenatal testing
US: United States
Cf-DNA: Cell-free DNA
SCD: Sickle cell disease
ACOG: American College of Obstetricians and Gynecologists
